# Supermarket Transaction Records In Dietary Evaluation – the STRIDE validation study.

**DOI:** 10.23889/ijpds.v8i3.2267

**Published:** 2023-09-11

**Authors:** Victoria Jenneson, Darren Greenwood, Graham Clarke, Timothy Rains, Becky Shute, Michelle Morris

**Affiliations:** 1 University of Leeds; 2 Supermarket retailer

## Introduction & Background

Supermarket transactions leave a digital footprint which offers insight into dietary habits. Use of transactions in nutrition research has increased, but these data are rarely validated. The STRIDE (Supermarket Transaction Records In Dietary Evaluation) study compares dietary estimates from supermarket transactions with self-reported intake from an online Food Frequency Questionnaire (FFQ).

## Objectives & Approach

Working with a large UK supermarket, loyalty card customers were recruited to one of four waves (accounting for seasonal dietary variation). Participants completed an online FFQ and consented to sharing their transaction records for one year during the study, and one year prior. The Bland-Altman method was used to calculate agreement and limits of agreement between transactions and intake for daily energy, sugar, total fat, saturated fat, protein and sodium (absolute and energy-adjusted).

## Relevance to Digital Footprints

Supermarket transactions are a form of digital footprints data with advantages over survey methods, with regards scalability and objectivity, for monitoring population-level diets.

## Results

1,788 participants from four UK regions gave consent. 686 participants who completed the FFQ and made purchases during the same period, were included for analysis. Participants were mostly female (72%), with a mean age of 56 years (SD 13). A regression equation for agreement is presented for estimating intake from purchases. Agreement for absolute measures was poor overall, but higher for single-person households and households reporting a higher proportion of total food purchases from the study retailer. Agreement was stronger for energy-adjusted nutrient estimates, particularly fat, with purchase records under-estimating the proportion of total energy intake from fat by just 2%.

## Conclusions & Implications

The STRIDE study found that household purchases from a single retailer were a poor proxy for individual-level nutrient intakes. However, close agreement on average energy-adjusted estimates suggests purchases are a good indicator of dietary composition. Supermarket transactions have utility for population dietary assessment, ecological studies, and identifying intervention targets based on dietary patterns. Digital footprint data from transactions can contribute to the design and monitoring of national and local-level interventions.

